# Study of Relationship Between Mode of Conception and Non-Specific Psychological Distress in Women Undergoing Noninvasive Prenatal Testing

**Published:** 2020

**Authors:** Nobuhiro Suzumori, Eri Takeda, Takeshi Ebara, Kyoko Kumagai, Yuki Sawada, Mayumi Sugiura-Ogasawara

**Affiliations:** 1- Department of Obstetrics and Gynecology, Nagoya City University Hospital, Nagoya, Japan; 2- Department of Occupational and Environmental Health, Nagoya City University Hospital, Nagoya, Japan

**Keywords:** ART, Depression, Infertility, NIPT, Prenatal diagnosis

## Abstract

**Background:**

Noninvasive prenatal testing (NIPT) has been performed worldwide to detect common fetal chromosomal aneuploidies.

**Methods:**

Pregnant women (n=3743) with advanced maternal age who visited Nagoya University for NIPT were enrolled in this study. The K6 mental stress scores, that is non-specific psychological distress scores were obtained by questionnaires which were administered pre-NIPT and postpartum. High K6 scores (≥10) indicate anxiety or depression. The K6 stress scores at pre-NIPT and postpartum were evaluated about the relationship between mode of conception and non-specific psychological distress using binomial logistic regression.

**Results:**

In general, 7.5% of pre-NIPT women (179/2393) and 5.1% of postpartum women (121/n) were found with high K6 scores. They also did not differ significantly based on maternal age, previous live birth, previous miscarriage, and mode of conception, *i.e.*, natural conception, artificial insemination with husband (AIH), or assisted reproductive technology (ART). Moreover, the prenatal K6 scores were not significantly higher than those at postpartum.

**Conclusion:**

Our present data suggest that mental distress in women undergoing NIPT during pregnancy and after birth has no statistical relationship with maternal age, previous live birth, previous miscarriage, or infertility treatment, and continuous mental care may help reduce mental distress in the postpartum period.

## Introduction

To detect common fetal chromosomal aneuploidies, noninvasive prenatal testing (NIPT) has been performed worldwide so far (1–3). A nationwide NIPT trial in Japan for trisomies 21, 18, and 13 has been conducted by the Japan NIPT consortium since April 2013 (4, 5). The test sensitivity and specificity of NIPT is much higher than those of conventional maternal serum screening for trisomies. Between April 2013 and March 2019, the Japan NIPT consortium has studied more than 50,000 test results in 73 medical institutions approved by the Japanese Association of Medical Sciences as clinical data.

Lately, the number of pregnancies at advanced maternal age using assisted reproductive technology (ART) has been increasing in Japan, and NIPT for fetal anomalies has raised concerns regarding the need for a more sensitive prenatal testing choice (6). In this paper, it was found that pregnant women who underwent NIPT tend to have relatively high scores for depression and anxiety (7), particularly in women (n=697) undergoing ART (8). Because the sample size of women undergoing NIPT was limited in the previous study, an attempt was made to reassess rate of fetal chromosomal abnormalities and the relationship between maternal age, previous live birth, previous miscarriage, or mode of conception, and non-specific psychological distress by binomial logistic regression model.

## Methods

Pregnant women (n=3743) with advanced maternal age who visited our university between June 2014 and December 2017 for NIPT were enrolled in this study. Their blood samples were collected and NIPT analysis was performed by massively parallel sequencing for fetal trisomies 13, 18, and 21 at Kazusa DNA Institute (Chiba, Japan) (6). The women confirmed their responses by questionnaires completed one month postpartum.

The K6 scores, which were considered representative of mental status, show how frequently the respondents experienced symptoms of non-specific psychological distress, which means feeling depressed and anxious (9). According to the Japanese validation study of K6 in the general population, performance of K6 in detecting anxiety disorders by the area under receiver operating characteristic curve (AUC) was excellent with values as high as 0.94 (95% confidence interval=0.88 to 0.99) (7). The self-administered questionnaires were assessed using a five-category scale (4=Always, 3= Often, 2=Sometimes, 1=Seldom, 0=Never), with a score range of 0–24. High K6 scores (≥10) indicate anxiety or depression (7, 8). The questionnaires were administered pre-NIPT (At approximately 10–12 weeks of gestation) and postpartum (One month after delivery) in women undergoing NIPT, excluding women with stillbirths.

Invasive prenatal procedures by amniocentesis were performed if the NIPT results were positive for trisomies 13, 18, or 21; all nonreportable results were confirmed by retesting or invasive testing using amniocentesis. The covariables were maternal age, previous live birth, previous miscarriage, and conception mode (*i.e*., natural conception, artificial insemination with husband (AIH), or ART including in vitro fertilization and embryo transfer (IVF-ET) and intracytoplasmic sperm injection (ICSI)).

The invasive prenatal procedures by amniocentesis were performed, and the characteristics and outcome of women who underwent NIPT, including prenatal, postnatal, and neonatal data were collected at one month postpartum. Statistical significance was determined by binomial logistic regression using IBM SPSS software version 23 (Chicago, IL, USA). Significant difference was determined at p≤0.05. This study was approved by the Research Ethics Committee of our University.

## Results

In this study, 3743 pregnant women with advanced maternal age, which is usually **defined** as **age** 35 or more for the **mother** at the time of delivery, visited our university for NIPT using massively parallel sequencing for fetal trisomies 13, 18, and 21. Of the 3743 women undergoing NIPT, 2393 women (63.9%), who confirmed their responses with questionnaires completed after fetal birth, were enrolled in this study. The mean age of participants was 38.4 years (Range: 34–47 years).

Table 1 shows the characteristics and pregnancy outcomes. No false negative cases were found in this study. Next, 548 pregnant women (22.9%) underwent ART (IVF-ET and ICSI-ET). Totally, 7.5% of pre-NIPT women (179/2393) and 5.1% of postpartum women (121/2393) were found with high K6 scores (≥10) (Table 2). They also did not differ significantly based on maternal age, previous live birth, previous miscarriage, and mode of conception, *i.e*., natural conception, AIH, or ART. In addition, the prenatal K6 scores were not significantly higher than those at postpartum. Also, fetal trisomies 13, 18, or 21 (n=37) confirmed by amniocentesis depended on the maternal age (OR of 1.38 per one year of age) (Table 2).

**Table 1.** The characteristics and pregnancy outcomes in our study population

		**Study population (n=2393)**
**Maternal age (years)**	Mean±SD	38.4±2.5, (34–55)
**Partner age (years)**	Mean±SD	39.3±4.6, (25–65)
**Marriage age (years)**	Mean±SD	33.1±4.2, (18–45)
**First pregnancy age (years)**	Mean±SD	35.9±3.8, (18–55)
**Previous live birth, n (%)**	0	1266 (52.9)
	1	860 (35.9)
	2	237 (9.9)
	3	26 (1.1)
	≤4	4 (0.17)
**Previous miscarriage, n (%)**	0	1680 (70.20)
	1	500 (20.89)
	2	151 (6.31)
	≤3	62 (2.6)
**Mode of conception, n (%)**	Natural conception	1524 (63.7)
	AIH	321 (13.4)
	ART (IVF-ET and ICSI)	548 (22.9)
**Maternal body mass index**	Mean±SD	21.0±3.0, (14.5–42.0)
**Gestational age at test (weeks)**	Mean±SD	13.7±1.0, (11.0–16.4)
**Trisomy, n (%)**	Trisomy 21	26 (1.1)
	Trisomy 18	10 (0.4)
	Trisomy 13	1 (0.04)
**Gestational age at delivery (weeks)**	Mean±SD	38.8±2.3, (22.0–43.0)
**Birth weight (*g*)**	Mean±SD	2973.4±503.5, (390–5400)
**Birth height (*cm*)**	Mean±SD	49.0±3.1, (20.0–58.0)
**IUFD/stillbirth, n (%)**		43 (1.8)

Note: ART: Assisted reproductive technology; AIH: Artificial insemination with husband; IVF-ET: In vitro fertilization and embryo transfer; ICSI: Intracytoplasmic sperm injection; IUFD: Intrauterine fetal death

**Table 2.** The study of the pre-NIPT, postpartum K6 high and fetal trisomy using binomial logistic regression analysis

	**Pre-K6 high (n=179)**			**Post-K6 high (n=121)**			**Fetal trisomy (n=37)**		

	**ORs**	**95% CI**	**p-value**	**ORs**	**95% CI**	**p-value**	**ORs**	**95% CI**	**p-value**
**Maternal age**									
	0.98	0.92–1.04	0.56	0.92	0.85–0.99	0.03	1.38	1.22–1.55	0.0001^*^
**Previous live birth (Yes/No)**									
	1.13	0.91–1.40	0.28	0.94	0.71–1.24	0.67	0.91	0.46–1.80	0.79
	1			1			1		
**Previous miscarriage (Yes/No)**									
	0.84	0.66–1.06	0.14	0.96	0.74–1.26	0.77	1.97	0.88–4.41	0.10
	1			1			1		
**Natural conception mode**									
	1			1			1		
**AIH**									
	0.85	0.46–1.58	0.61	0.72	0.37–1.41	0.34	0.79	0.17–3.71	0.76
**ART (IVF-ET and ICSI)**									
	0.84	0.47, 1.50	0.55	0.64	0.34–1.21	0.17	1.16	0.27–5.05	0.84

Note: ART: Assisted reproductive technology; AIH: Artificial insemination with husband; IVF-ET: In vitro fertilization and embryo transfer; ICSI: Intracytoplasmic sperm injection

## Discussion

The present study showed that 7.5% and 5.1% of women undergoing NIPT had high K6 score, and it is speculated that some women may have anxiety or depression during the pregnancy and post-partum periods, respectively. The high K6 score was not influenced by AIH or IVF-ET. It was also shown that fetal trisomies depended on maternal age but not the mode of conception.

Our previous study presented that even if women with NIPT did not experience mental distress before NIPT, those who conceived through ART (IVF-ET and ICSI-ET) may experience mental stress during the postpartum period (8). Stanhiser and Steiner reported that couples with infertility struggled in coping with the psychosocial aspects of ART (10). Although no relationship was found between infertility treatment and non-specific psychological distress using binomial logistic regression, information before and after NIPT must be provided to health care professionals for genetic counseling.

Several lines of evidence suggest that the negative predictive value is almost the same between NIPT and conventional serum screening test (2, 3). In this study, no false negative cases were found, so that NIPT was assumed to be appropriate for fetal screening in women with advanced maternal age. Conventional serum screening using ultrasounds is not common in Japan because of the limited number of co-medical professional workers, however, NIPT is useful for fetal chromosomal screening in the country.

The relationship between advanced maternal age and trisomy has been identified for over 50 years and is one of the most important etiological factors associated with any human genetic disorder (11). The risk of trisomy in a conception rises from approximately 2%–3% for women in their 20 *s* to ≥30% for women in their 40 *s*. Our present data also suggested that the increased risk of trisomies 13, 18, and 21 was recognized due to advanced maternal age, and the OR was 1.38 per one year of age in pregnant women.

Because NIPT can be performed at its current pace, it will provide comprehensive prenatal information (12). Although it increases patient autonomy and comfort, this revelation of fetal information may also increase patient anxiety or generate undeserved outcomes including selective abortion. Our present data suggest that women, regardless of infertility treatment, after undergoing NIPT tend to have psychological distress postpartum. Couples undergoing NIPT must be continuously supported to reduce psychological distress.

NIPT is now increasingly used for screening of sex chromosome aneuploidies, microdeletions, and multiple Mendelian monogenic disorders (13, 14). Genetic counseling to postpartum women including consultations with mental healthcare professionals and psychological support must be offered (15, 16). Additional assessments may also be needed if continuous mental care helps reduce their mental distress while caring for their children.

## Conclusion

Pregnant women with advanced maternal age for NIPT were enrolled, and the K6 mental stress scores were administered pre-NIPT and post-partum. High K6 scores (≥10) indicate anxiety or depression. In general, 7.5% of pre-NIPT women and 5.1% of postpartum women were found with high K6 scores. They did not differ significantly based on maternal age, previous live birth, previous miscarriage, and mode of conception, *i.e*., natural conception, AIH, or ART. Our findings suggest that mental distress in women with NIPT during pregnancy and after birth has no statistical relationship with maternal age, previous live birth, previous miscarriage, or infertility treatment.
